# Optimized high-throughput microRNA expression profiling provides novel biomarker assessment of clinical prostate and breast cancer biopsies

**DOI:** 10.1186/1476-4598-5-24

**Published:** 2006-06-19

**Authors:** Michael D Mattie, Christopher C Benz, Jessica Bowers, Kelly Sensinger, Linda Wong, Gary K Scott, Vita Fedele, David Ginzinger, Robert Getts, Chris Haqq

**Affiliations:** 1 UCSF Comprehensive Cancer Center, Department of Urology, San Francisco, California 94115, USA; 2 Genisphere Inc., Hatfield, Pennsylvania 19440, USA; 3 Applied Biosystems, Foster City, California 94404, USA; 4 Buck Institute for Age Research, Novato, California 94945, USA

## Abstract

**Background:**

Recent studies indicate that microRNAs (miRNAs) are mechanistically involved in the development of various human malignancies, suggesting that they represent a promising new class of cancer biomarkers. However, previously reported methods for measuring miRNA expression consume large amounts of tissue, prohibiting high-throughput miRNA profiling from typically small clinical samples such as excision or core needle biopsies of breast or prostate cancer. Here we describe a novel combination of linear amplification and labeling of miRNA for highly sensitive expression microarray profiling requiring only picogram quantities of purified microRNA.

**Results:**

Comparison of microarray and qRT-PCR measured miRNA levels from two different prostate cancer cell lines showed concordance between the two platforms (Pearson correlation R^2 ^= 0.81); and extension of the amplification, labeling and microarray platform was successfully demonstrated using clinical core and excision biopsy samples from breast and prostate cancer patients. Unsupervised clustering analysis of the prostate biopsy microarrays separated advanced and metastatic prostate cancers from pooled normal prostatic samples and from a non-malignant precursor lesion. Unsupervised clustering of the breast cancer microarrays significantly distinguished ErbB2-positive/ER-negative, ErbB2-positive/ER-positive, and ErbB2-negative/ER-positive breast cancer phenotypes (Fisher exact test, p = 0.03); as well, supervised analysis of these microarray profiles identified distinct miRNA subsets distinguishing ErbB2-positive from ErbB2-negative and ER-positive from ER-negative breast cancers, independent of other clinically important parameters (patient age; tumor size, node status and proliferation index).

**Conclusion:**

In sum, these findings demonstrate that optimized high-throughput microRNA expression profiling offers novel biomarker identification from typically small clinical samples such as breast and prostate cancer biopsies.

## Background

MicroRNAs (miRNA) are a class of small non-coding RNAs encoded in the genomes of animals and plants [1-4] that play a role in targeting messages of protein-coding genes for cleavage or translational repression [5,6]. The active miRNA products ~22 nt in length are formed from larger 60–110 nt hairpin precursor transcripts that serve as substrates for the dsRNA endoribonuclease Dicer [7,8]. The mature miRNAs formed by Dicer cleavage are short dsRNA molecules, one strand of which is incorporated into the ribonucleoprotein complex RISC (RNA induced silencing complex) for subsequent targeting to mRNAs [9]. Complementary mRNA sequences are inactivated by cleavage in a fashion similar to RNAi, while pairing with partially complementary sequences in the 3' UTR of target mRNAs can either repress translational efficiency or induce transcript decay [10-12]. Present estimates suggest that nearly a third of all cellular transcripts may be regulated by the few hundred human miRNAs currently known to exist [1].

Recently, miRNAs have been shown capable of distinguishing the different tissue developmental lineages and differentiation states of various human malignancies [13], including breast cancer [14]. In particular, comparison of normal and malignant breast tissue has revealed that a small subset of deregulated miRNAs (including mir-125b, mir-145, mir-21, and mir-155) can be identified that unequivocally distinguish normal from malignant breast tissue, as well as other differentially expressed miRNAs that appear to correlate with breast cancer histopathologic features such as tumor size, nodal involvement, proliferative capacity and vascular invasiveness [14]. While specific miRNAs may be postulated to regulate the expression of genes involved in receptor networks known to drive breast cancer progression, miRNA profiling has not yet been shown capable of independently identifying breast cancer phenotypes clinically defined by the overexpression of ErbB2 and/or estrogen receptor (ER) proteins. Nonetheless, the provocative early observations of miRNAs expressed in human breast cancer are stimulating broad interest in the possibility that miRNA profiles represent a promising new class of cancer biomarkers. However, progress in more widespread evaluation of miRNAs as potential cancer biomarkers remains limited by current miRNA assay methods and platforms.

The most extensively used approaches to miRNA identification and quantification include cloning, northern blot, and microarray-based methods. Cloning methods can require hundreds of micrograms of total RNA [15-17], while northern blot methods typically use 10–30 μg per analysis [10,16,17]. These methods are problematic in that they are not high-throughput and they consume far more tissue-derived RNA than is typically available from a clinical biopsy sample. Several previously described high-throughput microarray approaches, as well as other platforms, also utilize microgram or greater quantities of total RNA [13,18-23]. Depending on the number of core biopsies taken per patient, as well as the number and thickness of tissue sections made available, total sample yield from a typical core biopsy may be 200 ng or less of total RNA. Thus, in order to perform high-throughput microarray-based miRNA expression profiling on typical clinical biopsy samples, further optimization of miRNA analysis is needed. We adapted a previously described method [24] for the amplification and labeling of miRNAs for microarray-based analysis and compared expression levels to levels detected by a TaqMan^® ^quantitative RT-PCR platform. Our high-throughput miRNA expression profiling approach was ultimately applied to test the ability of differentially expressed miRNAs to distinguish malignant from non-malignant prostate cancer samples, and to blindly classify breast cancers in accordance with their clinically defined ErbB2 and ER status as well as potentially identify miRNA signatures associated with ErbB2 and ER phenotypes.

## Results

### Reproducibility of miRNA profiling by microarrays and their validation by qRT-PCR

**Figure 1:**
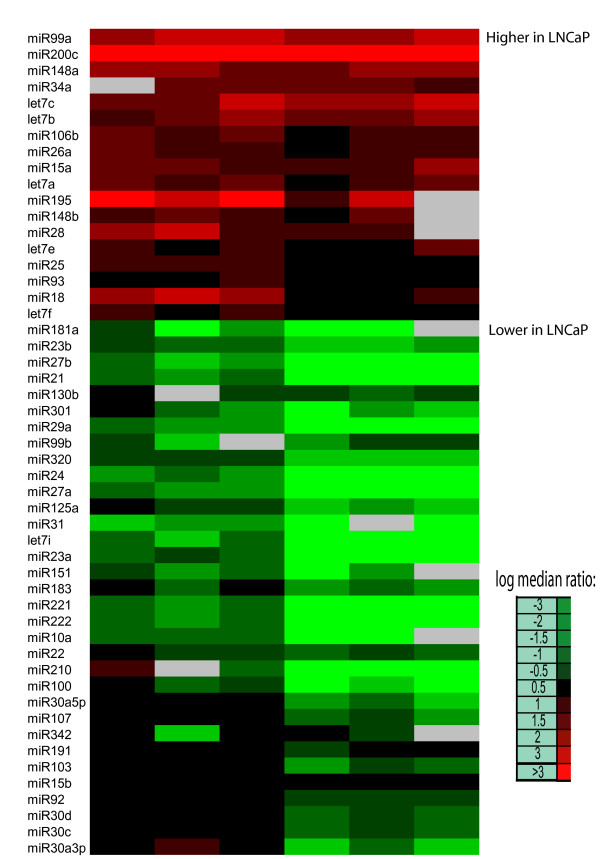
**Comparison of microRNA expression in PC3 and LNCaP cells by microarray analysis**. A heat map of replicate experiments (N = 6) that displays the relative Cy3/Cy5 ratios of microRNAs expressed in PC3(Cy3) vs.LNCaP(Cy5) cells. Expression levels on individual arrays were calculated by averaging the duplicate spots for each miRNA. MicroRNAs are listed in order of significance as determined by SAM analysis. MicroRNA expression is displayed as higher (red) or lower (green) in LNCaP cells in comparison to PC3 cells.

To examine the reproducibility of the labeling procedure (Additional file 1) equal amounts of miRNA (150 ng, derived from about 1.0 microgram of totalRNA) from the human prostate cell line PC3 was labeled with Cy3 and Cy5 in parallel reactions and hybridized to sense strand-spotted arrays. A comparison of the Cy3 and Cy5 labeled PC3 miRNA signal intensities indicated highly reproducible labeling of the same sample in both channels (R^2 ^= 0.99, Additional file 2). In further examination of reproducibility, duplicate labeling reactions were performed using 100 ng of normal (Cy3) and tumor (Cy5) miRNA samples for hybridization to the arrays. Comparison of the Cy3/Cy5 ratios between the two representative arrays showed a high degree of concordance (R^2 ^= 0.94, Additional file 2). Multiple replicates of labeled PC3 vs. LNCaP miRNA revealed distinct expression patterns between the two cancer cell lines that were highly consistent; Figure 1 displays the averages of the duplicate spots for each miRNA probe. Due to the high concordance between replicate spots, averaging of the duplicates had little to no effect on the microarray analysis results. Successful labeling and expression profiling was achieved with as little as 10 ng of enriched miRNA.

**Figure 2:**
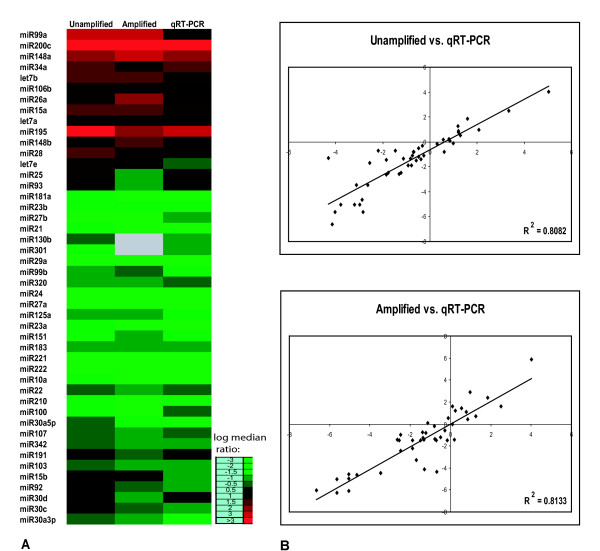
**Comparison of microRNA expression measurements by microarray and RT-PCR**. **A) **A heat map comparing the average fold-changes in microRNAs with significantly higher (red) or lower (green) expression in LNCaP cells in comparison to PC3 cells as determined either by Taqman^® ^qRT-PCR or microarray analysis (unamplified and amplified). Genes included in the figure are those found to be significantly different by one or both methods. Mir-130b and miR-301 were not found to be significantly different in amplified samples by SAM analysis. **B) **Pearson correlation scatter plots of comparisons of array data to qRT-PCR for amplified and unamplified miRNA.

Microarray analyses identified differentially expressed miRNAs when comparing the expression patterns of the hormone sensitive LNCaP and hormone insensitive PC3 prostate cancer cell lines. To verify the accuracy of these microarray results we measured expression of individual miRNAs by a real-time quantitative TaqMan qRT-PCR method designed to detect mature miRNA sequences [25]. Figure 2 displays a comparison of qRT-PCR and microarray results for those microRNAs differentially expressed between LNCaP and PC3 cells. Analysis of the fold changes for these miRNAs showed good concordance (R^2 ^~ 0.81) between the microarray and RT-PCR platforms. We did not observe any cases where the direction of the change in miRNA expression was discordant between the two platforms; the only discordance observed was for let-7 miRNA family members which were found upregulated by microarray measurement and not significantly changed by qRT-PCR measurement (Additional file 3).

### Sensitivity and reproducibility of the miRNA amplification procedure

**Figure 3:**
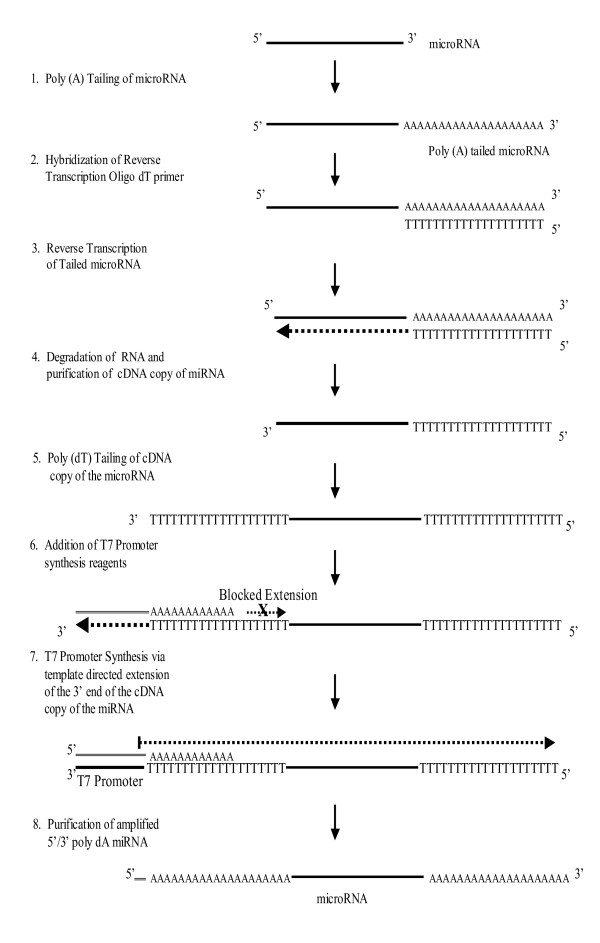
**Schematic of microRNA amplification procedure**. A poly A tail is first added at the 3' end of microRNAs. Reverse transcription performed using a primer consisting of an oligo dT and a capture sequence. Next, the first strand cDNA is tailed with dTTP using terminal deoxynucleotidyl transferase on the 5' end, followed by annealing of a T7 template oligo to the 3' tail of the cDNA. Klenow enzyme fills in the 3' end of the first strand cDNA to produce a double-stranded T7 promoter. The T7 template contains a blocker to prevent second strand synthesis. An *in vitro* transcription reaction with T7 RNA polymerase is then performed. Amplified products are then labeled using the method previously mentioned starting at the reverse transcription step.

The linear amplification technique developed for miRNA expression profiling in cases where starting sample amount is very limited is schematically shown in Figure 3, and represents an adaptation of a previously reported method[24]. Five different input amounts of miRNA were titrated into the amplification protocol to determine linearity of the yield; as shown in Table 1 the yields from this titration produced an excellent linear regression correlation (R^2 ^= 0.95). Amplification of miRNA by this method has been successfully performed on as little as 250 pg of enriched miRNA (~2 ng total RNA), an amount of RNA easily obtained from needlecore biopsies or even fine needle aspirates.

**Table 1:** Titration of amplified miRNA

**miRNA input (ng)**	**Yield (ng)**
0.5	2664
1	2791
3	8165
9	22736
27	40340

To examine the reproducibility of the amplification procedure, 500 pg of enriched miRNA isolated from a prostate needle core biopsy was amplified in duplicate. Equal amounts (750 ng) of both amplifications were labeled with either Cy3 or Cy5 and hybridized to an array. Comparison of the respective signal intensities showed highly reproducible amplification and labeling (R^2 ^= 0.988, Supplemental file 2). Additionally, the fidelity of the amplification procedure was assessed by comparing microarrays of unamplified and amplified miRNA samples derived from tumor and normal (adjacent to tumor) tissue from the same patient. For the amplified samples, 500 pg of enriched tumor and normal miRNA was amplified, and 1 μg from each reaction was labeled with either Cy3 (tumor) or Cy5 (normal). Parallel labeling reactions were performed using 150 ng of unamplified miRNA from the same samples. Comparison of the differential expression of amplified to unamplified miRNAs showed that the amplification procedure produced excellent fidelity in miRNA representation (R^2 ^= 0.875). As well, microarray comparison of amplified PC3 and LNCaP miRNAs produced a similar degree of concordance with unamplified samples (R^2 ^= 0.8133), and also with qRT-PCR measurements (R^2 ^= 0.8082), as shown in Figure 3.

### MicroRNA expression profiling distinguishes malignant from non-malignant prostate tissue

**Figure 4:**
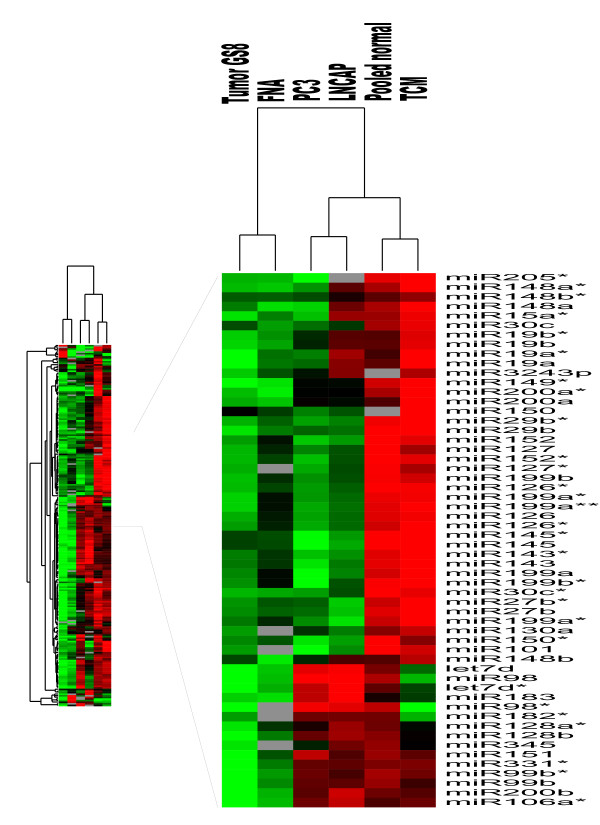
**Unsupervised hierarchical cluster of amplified prostate needle core biopsies**. Expression patterns of amplified miRNA from a small set of clinical prostate specimens. Cluster analysis of the microarrays displayed shows a cluster on the left consisting of an advanced needle core biopsy of an advanced prostatic tumor (Gleason score 8) and a fine needle aspirate of a prostatic lymph node metastasis (FNA), a cluster in the middle consisting of PC3 and LNCaP prostate tumor cell lines, and a cluster on the right consisting of a pooled normal adjacent to tumor sample and another sample consisting of transitional cell metaplasia (TCM), an early precursor lesion.

Microarray profiling of miRNAs isolated and amplified from prostate needle core biopsy specimens was performed to demonstrate the utility of this methodology on small clinical samples. Pooled normal prostate miRNA was made from total RNA individually extracted from 10 different needle core prostate biopsies showing normal histology (average patient age, 63 years). A total of 20 ng pooled total RNA from the 10 biopsies was enriched for miRNA (as described in Methods) and amplified. The pooled and amplified normal prostate miRNA was compared against enriched and amplified miRNA samples (10 ng) produced from two abnormal prostate needle core biopsies (one showing an advanced prostate cancer and another showing a non-malignant precursor lesion referred to as transitional cell metaplasia), and a fine needle aspirate of a prostate cancer metastasis to a supraclavicular lymph node. As shown in Figure 4, unsupervised clustering analysis performed on the different microarrayed miRNA samples (pooled normal prostate, two needle core cancer biopsies, fine needle node aspirate, and PC3 and LNCaP reference samples) unequivocally discriminated the prostatic cancer tissues from the pooled normal samples and non-malignant precursor lesion.

### MicroRNA expression profiling identifies clinically relevant breast cancer phenotypes

Overexpression of either the ErbB2 or ER receptors usually results in clinically distinct breast cancer phenotypes, which are detectable by gene expression profiling [26]. However, breast cancer overexpression of both ErbB2 and ER is not uncommon and is associated with a different and clinically troublesome phenotype [27]. A panel of 20 different breast cancer samples was chosen to represent these three common phenotypes (9/20, ErbB2-positive/ER-negative; 4/20, ErbB2-positive/ER-positive; 7/20, ErbB2-negative/ER-positive) and was blindly analyzed for miRNA expression levels by microarray profiling. Since a sample of pooled normal mammary gland total RNA was not available, all tumor miRNA samples were normalized to miRNA prepared from a single age-matched sample of normal mammary gland tissue. An ErbB2-positive/ER-negative breast cancer cell line, SKBr3, was also used for reference. Comparable with regard to histology and stage, the cancers had also previously been annotated with regard to progesterone receptor (PR) overexpression (11/20), tumor cell proliferation index, and the presence of any p53 DNA mutations (see Additional file 4 for clinical annotation). Using 500 ng of total RNA from all samples, 10 ng of miRNA was amplified and the resulting amplification product labeled and array hybridized.

**Figure 5:**
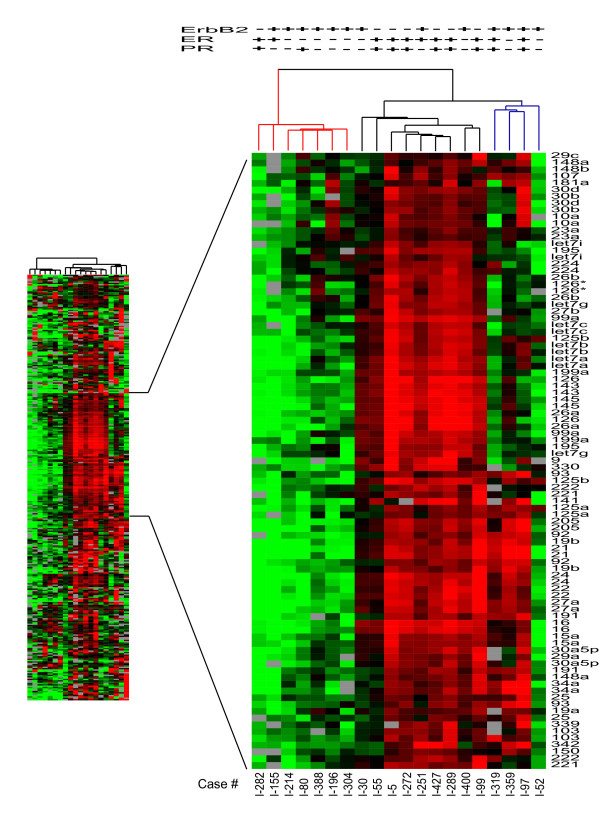
**Unsupervised hierarchical cluster of breast tumor samples**. The tree was generated by cluster analysis of all 20 breast tumor tissue samples and displays the clinical pathologic features for ErbB2, estrogen receptor (ER), progesterone receptor (PR), and p53 (w = wild type, m = mutant). Cluster analysis revealed three distinct branches of the dendrogram. Duplicate spots for each probe are displayed and were not averaged for this analysis to demonstrate the consistency of expression values between duplicate spots. The probe sequence for mir-126* is distinctively different from mir-126.

As shown in Figure 5, unsupervised hierarchical clustering of the resulting miRNA profiles revealed 2 or 3 major dendrogram groups. Unblinding this analysis to the clinical annotation of each sample revealed that the 2 mostprominent dendrogram groups were primarily distinguished by lack of ER-positive (2/7) or PR-positive cancers (2/7) in the first group and the presence of ER-positive (9/13) or PR-positive cancers (8/13) in the second group; these differences did not quite achieve statistical significance (Fisher Exact test, p = 0.16). In contrast, the 3 major dendrogram arms were primarily distinguished by their different proportions of ErbB2-positive cancers, with the first showing almost exclusively ErbB2-positive cancers (6/7), the second showing very few ErbB2-positive cancers (3/9), and the smallest and most heterogeneous of these arms showing only ErbB2-positive cancers (4/4). These ErbB2 differences between the 3 major dendrogram arms proved statistically significant (Fisher's Exact test, p = 0.03); and in consideration with ER status these 3 groups suggest that they may be generally representative of ErbB2-positive/ER-negative, ErbB2-negative/ER-positive, and ErbB2-positive/ER-positive breast cancers, respectively. When the miRNA levels detected in the ErbB2-positive breast cancers were averaged apart from the other breast cancers and compared to the 67 different miRNAs found expressed in the ErbB2-positive breast cancer cell line, SKBr3, a surprising concordance was observed (Spearman rank order correlation, R_s _= +0.43, n = 67, p < 0.001), suggesting that this cell line model of ErbB2-positive breast cancer closely reflects the miRNA expression profile found in clinical samples of ErbB2-positive breast cancers (Supplement file 4). No significant differences were found between any of the three breast cancer subgroups (Fisher's Exact test, p > 0.10) when evaluated by any of other clinically relevant parameters (patient age,% tumor cells, tumor size, nodal status, grade, proliferation index, p53 status; Additional file 5).

**Figure 6:**
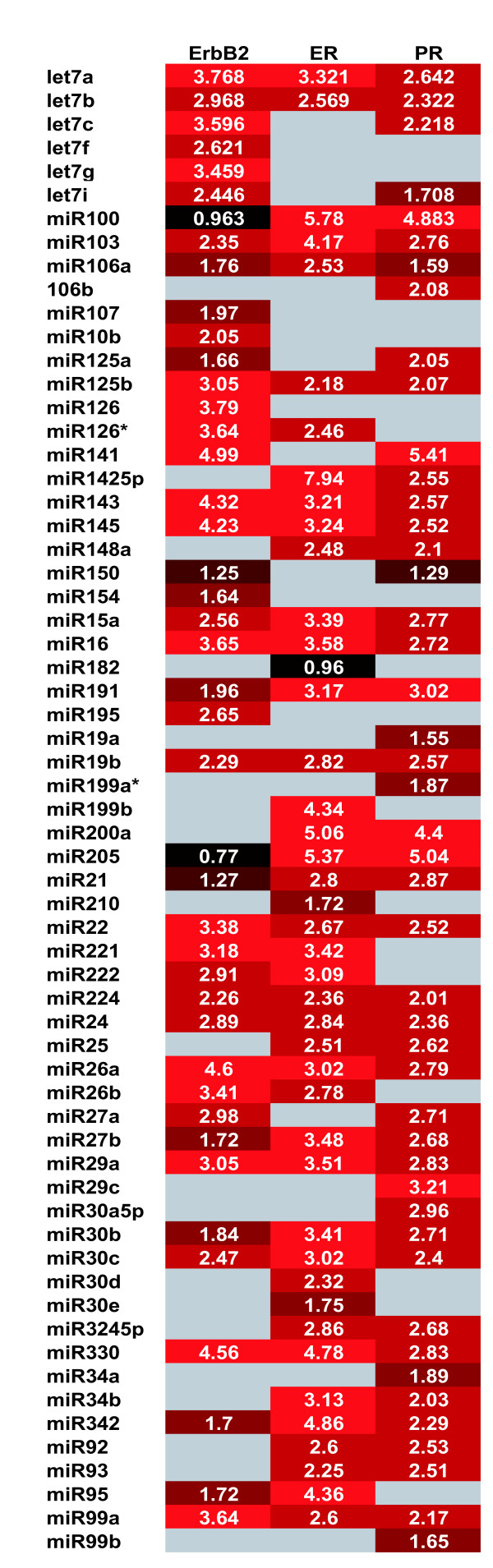
**Comparison of miRNA expression profiles associated with receptor status**. **A) **Heat map of genes found significantly different according to SAM analysis. Map displays genes significantly higher in ErbB2-negative vs. ErbB2-positive tumors, genes significantly higher in ER-positive vs. ER-negative tumors and genes significantly higher in PR-positive vs. PR-negative tumors.

SAM analyses were performed on the entire set of 20 breast cancers to identify potential differences in miRNA signatures between the following phenotypic groups: ErbB2-negative vs. ErbB2-positive, ER-positive vs. ER- negative, PR-positive vs. PR-negative, and mutated vs. wild type p53. No significant differences were found according to p53 status, although this analysis was obviously limited by the few cases containing mutated p53. For ErbB2 status, however, it was found that 43 miRNAs were significantly higher in ErbB2-negative as compared to ErbB2-positive breast cancers. Likewise, 43 miRNAs were significantly higher in ER-positive vs. ER-negative cancers, while 46 miRNAs were higher in PR-positive vs. PR-negative cancers. Comparison of the three signature profiles associating with ErbB2, ER and PR status (Figure 6) revealed many genes in common and a more restricted subset of miRNAs specific to ErbB2 status (*let-7f, let-7g, miR-107, mir-10b, miR-126, miR-154 and miR-195*) and a smaller subset specific to ER/PR status (*miR-142-5p, miR-200a, miR-205 and miR-25*).

## Discussion

The amplification, labeling and microarray methodology described herein permits sensitive, accurate, and high-throughput microRNA profiling of small clinical sample specimens. Faithful amplification was achieved using as little as 250 pg of enriched miRNA (~2 ng total RNA), an amount easily obtainable from needle core biopsies or even fine needle aspirates. The amplified miRNA demonstrated good replication fidelity in comparison to unamplified RNA, indicating that all species of miRNAs were comparably amplified and suggesting that this procedure is superior to alternative methods such as blunt-end ligation of adaptors for PCR amplification, which suffer from questionable RNA ligase reliability. Use of a dendrimer-based labeling system [28] in combination with miRNA amplification is key to achieving high miRNA detection sensitivity. The dendrimer approach has an advantage over direct and other indirect labeling methods because the multiple fluorophores are an integral part of the dendrimer and do not have to be incorporated during cDNA preparation; this avoids inefficient hybridization of the cDNA to the array by incorporation of fluorescent dye conjugates into the reverse transcript [29]. Since each branched dendrimer has approximately 900 attached fluorophores, miRNA signal intensity is amplified and 10–20 fold less input miRNA is required as compared to other previously reported techniques [28,30,31].

Results using this amplification and labeling methodology in conjunction with the microarray platform were validated by comparison with Q-RT-PCR measurement of miRNA levels. Only the levels of selected let-7 family members showed differences between microarray and RT-PCR quantitation. Since different let-7 family members may differ in sequence by only one nucleotide, there is some question regarding how accurately probes can distinguish such similar family members. In our previous experiments let-7 amicroarray levels were in agreement with northern blot results [32]; as well, microarray results for other miRNAs showing very similar sequences did not appear discordant with qRT-PCR results. Of note, the qRT-PCR method used here (Applied Biosystems) discriminates between single-base pair differences using gene-specific hairpin primers for the initial reverse transcription reaction. Additional specificity is achieved with the type of primers used in the qRT-PCR reaction. Using hairpin primers, the qRT-PCR method only detects mature miRNAs. Thus, we cannot rule out the possibility that immature forms of miRNA also hybridized to the probes on the microarrays, thereby inflating expression levels relative to those measured by qRT-PCR. However, based on our previous studies and those of others [32,33], the relative levels of precursor miRNAs are very low in comparison to mature miRNA forms. We acknowledge that normalization of qRT-PCR data by any particular miRNA(s) can skew the results in either direction. In the absence of any current consensus in the field regarding appropriate "housekeeping gene equivalents" in miRNA enriched fractions, we have since moved toward other statistical approaches such as scale factor normalization. Regardless of the normalization approach for the qRT-PCR data, there would not be a large impact on the correlation of expression values between microarray and qRT-PCR platforms. Northern blot confirmations in our subsequent studies [32] have also been consistent with our microarray results, giving confidence about results we report here, with the limitations of let-7 family member measurements due to the design of the array platform at the time these studies were conducted. Developing high-throughput microarray platforms that achieve highly sensitive and accurate discrimination of all miRNAs, including the most homologous of paralogs, remains a technical challenge, although recent studies suggest that further microarray detection specificity may be achieved by employing locked nucleic acid (LNA) modified oligonucleotides [21,34,35]. Improvements to the TaqMan miRNA assays and microarray platforms that have been made since the experiments described here are likely to further improve concordance between the detection platforms.

Microarray analysis of prostate biopsy samples and a panel of breast cancer samples served to validate the feasibility and utility of this new high-throughput methodology of miRNA profiling, proving it to be sufficiently sensitive and accurate for routine use on small clinical specimens such as those typically obtained by core biopsies or even fine needle aspiration. The informative nature and potential biomarker utility of the miRNA profiles detected in these clinical samples was revealed by unsupervised clustering analysis blinded to the samples' clinical annotations. The prostate biopsy miRNA profiles clearly discriminated between the malignant and non-malignant samples, while the breast cancer miRNA profiles discriminated clinically relevant breast cancer phenotypes.

The panel of 20 breast cancer samples was chosen *a priori *to represent three clinically important breast cancer subtypes, defined by ErbB2 and ER receptor status. Modern breast cancer treatments are based on these two validated biomarkers since ErbB2-positive breast cancers are treated with the ErbB2-targeted antibody, trastuzumab (Herceptin^©^), while ER-positive breast cancers are treated with either antiestrogens or estrogen-ablating aromatase inhibitors. At least a third of all ErbB2-positive breast cancers are also ER-positive, and because this breast cancer subgroup appears more refractory to all forms of endocrine therapy [27], it has attracted considerable attention among basic and clinical breast cancer investigators. ErbB2-positive and ER-positive breast cancers have been shown to exhibit significantly different gene expression profiles[26], however, gene expression studies to date have failed to discriminate ErbB2-positive/ER-positive breast cancers from either ErbB2-positive or ER-positive subgroups. It is therefore of great interest that unsupervised clustering of the miRNA profiles from the 20 breast cancer samples studied here clearly discriminated at least two subsets of ErbB2-positive breast cancers, one that is largely ErbB2-positive and ER-negative, and another more remotely related group of ErbB2-positive cancers with miRNA features more typical of ER-positive breast cancers. Additional breast cancer samples must be studied to conclusively identify a profile of miRNAs that define this clinically important subset of ErbB2-positive/ER-positive breast cancers. The important possibility that microRNA signatures may prove to be novel cancer biomarkers is apparent from this study's preliminary finding that unique sets of miRNAs are associated with breast cancers currently defined by their ErbB2 status (*let-7f, let-7g, miR-107, mir-10b, miR-126, miR-154 and miR-195*) or their ER/PRstatus (*miR-142-5p, miR-200a, miR-205 and miR-25*). Several of the breast cancer miRNAs identified in the present microarray analysis [10b, 21, 34, 125a, 125b, 126 145] were also found to be deregulated in a recent miRNA survey of a phenotypically more diverse breast cancer panel [14].

In sum, the present study has demonstrated the feasibility and utility of measuring miRNA profiles from clinically relevant biopsy samples using an optimized high-throughput microarray assay platform. Application of this methodology to the analysis of prostate and breast cancer biopsy samples suggests that specific miRNA expression signatures may be identified which, upon further evaluation, may prove to have important diagnostic, prognostic or predictive clinical value as cancer biomarkers.

## Methods

### Cell lines, breast tissue samples, and isolation of miRNAs

Initial methods development utilized cancer cell lines that were readily available in our lab. The prostate cancer cell lines, PC3 and LNCaP, and the breast cancer cell line, SKBr3, were grown at 37°C at 5% CO2 in RPMI-1640 media (Sigma) supplemented with 10% FBS and 100 U/ml penicillin and 100 μg/ml streptomycin. RNA obtained from these cancer cell lines was enriched for microRNAs using the mirVana miRNA Isolation Kit from Ambion (Austin, TX) according to manufacturer recommendations. Total RNA was Trizol (Invitrogen, Carlsbad, CA) extracted from 20 cryobanked primary breast cancer biopsies (-80° C, Bari, Italy), clinically annotated only for patient age (average 55 years, range 29–79 years), tumor state (stage I or II), histology (invasive ductal, grade, >50% malignant cellularity), ErbB2 (gene copy amplification), ER/PR (nuclear protein overexpression) positivity, proliferation index (Ki-67/MIB-1) and p53 mutation (exon 5–8) status. Similarly, total RNA was Trizol extracted from 15 cryobanked prostate core biopsy samples (UCSF Tissue Core) clinically annotated for patient age (average 63 years, range 52–70), histology (tumor grade, % malignant cellularity), and PSA. Control tissue used for comparison against the breast cancer samples was commercially obtained (Ambion, Austin, TX) and consisted of histologically normal breast tissue resected from a 55 year old woman; the commercially obtained (Ambion) control tissue used for comparison against the prostate cancer samples consisted of histologically normal prostate tissue resected from a 79 year old male. MicroRNAs were isolated from 500 ng total RNA extracted from normal or malignant breast tissues and from ≤100 ng total RNA extracted from the prostate biopsies, using the mirVana miRNA Isolation kit with their modified protocol for isolation of small RNAs. All microRNA measurements and unsupervised hierarchical clustering analyses were performed blinded to all annotated clinical features.

### Microarray methods

Printing, post-processing, and analysis of the microarrays was performed as described [36]. An oligonucleotide microarray was constructed containing all of the annotated human microRNA genes in the miRNA Registry [37]. Oligonucleotide probes identical to the sense orientation of the mature human miRNA sequences were synthesized by Operon Biotechnologies (Huntsville, AL) and duplicate spotted in 3X SSC on Gold Seal microslides (Becton Dickinson, Bedford, MA) coated with poly-l-lysine using a linear servo arrayer at the UCSF Core Facility for Genomics and Proteomics. Arrays were printed with probes consisting of a tandem repeat or dimer of the mature sequences (Additional file 6). In regard to oligonucleotide probe design for microarrays, others have demonstrated that increased sensitivity for microarray profiling can be gained by spotting probes that consist of multimers of the mature miRNA sequences [38]. At the time of printing the arrays contained all of the annotated human microRNA genes in the miRNA Registry as of November, 2004 (approximately 200). Some experiments used to validate the amplification method were performed using anti-sense arrays spotted and hybridized as previously described [38]. Data were submitted to the GEO database (accession numbers GSE4572, GSE4574 and GSE4589).

### Amplification, labeling and hybridization of microRNAs

MicroRNAs were amplified using the SenseAmp Plus amplification protocol [39] for miRNA from Genisphere (Hatfield, PA). The amplification procedure produces a "sense" copy of the RNA with short poly A sequences on both the 3' and 5' ends. Amplifications were performed with as little as 250 pg of enriched miRNA (~2 ng total RNA). For amplification input titration experiments five different amounts of miRNA (0.5–27.0 ng) were used. The resulting sense RNAs were quantitated using the Ribo Green RNA Quantitation kit from Molecular Probes (Eugene, OR). Unamplified samples enriched for miRNA and amplified miRNA samples were labeled with the Array900 miRNA kits from Genisphere according to the manufacturer protocol (Additional file 1). Briefly, 10–200 ng of enriched miRNA was used in the initial tailing reaction using *E. coli *Poly A Polymerase. The tailed miRNA was reverse transcribed using an oligo dT primer that incorporates a capture sequence tag on the miRNA. Two tagging sequences/primers were used in these experiments, one for binding to Cy3 labeled dendrimers [5'-TTCTCGTGTTCCGTTTGTACTCTAAGGT GGA-T(17)-3'] and a second for binding Cy5 labeled dendrimers [5'-ATTGCCTTGTAAGC GATGTGATTCTATTGGA-T(17)-3']. A hybridization mix containing the Cy3 and Cy5 tagged miRNA was hybridized to the array under a glass cover slip at 46°C for 16 hours in a Hybex hybridization oven (SciGene, Sunnyvale, CA). After washing the arrays a second hybridization using Cy3 and Cy5 labeled dendrimers having complementary sequences to the tags was run for 4 hours at 60°C to generate the fluorescent signal. Specific conditions used in amplification and labeling experiments are described in further detail in the Results section.

### Quantitative RT-PCR measurement of microRNAs

MicroRNA expression was also measured using a pre-release version of the TaqMan miRNA quantitative PCR assay from Applied Biosystems (Foster City, CA) that has been previously described [25]. Expression levels of all the annotated human miRs as of September, 2004 were analyzed using real-time TaqMan RT-PCR with the ABI PRISM 7900 instrument (Applied Biosystems, Foster City, CA). Briefly, cDNA was made from enriched miRNA in 15-μL reactions (1 ng/μl final concentration) using (Multiscribe) MuLV reverse transcriptase and specific primers for each miRNA. The cycle parameters for the RT reaction are 16°C for 30 minutes, 42°C for 30 minutes, 85°C for 5 minutes, hold at 4°C. The PCR reaction mix consists of the RT product, Taqman 2X Universal PCR Master Mix and the appropriate 5X MicroRNA Assay Mix containing primers and probe for the miRNA of interest. Cycle parameters for the PCR reaction are 95°C for 10 minutes (AmpliTaq Gold enzyme activation), followed by 40 cycles of a denaturing step at 95°C for 15 seconds and an annealing/extension step at 60°C for 60 seconds. All reactions were run in triplicate. Due to the fact that the samples used for these experiments were enriched for microRNA, it was not possible to normalize expression to the typical housekeeping genes as is done for mRNA expression. Previous experiments during development of the microRNA TaqMan assays demonstrated that let-7 and miR-16 share similar and highly abundant expression between various cell lines (see Additional file 7 for expression in PC3 and LNCaP cells). The expression of each miRNA relative to let-7 and miR-16 was determined using the ΔΔCt method. The threshold cycle (Ct) is defined as the fractional cycle number at which the fluorescence passes the fixed threshold. For our experiments four replicates each of LNCaP and PC3 were run in triplicate, two of which were normalized to miR-16 and the other two were normalized to let-7. Average fold differences were calculated by normalizing the relative expression (ΔΔCt values) in the LNCaP cell line to that in the PC3 cell line. Average fold differences below 0.5 or above 2.0 were considered to represent a significant difference between the two cell lines.

### Bioinformatic and statistical analyses

Cy3 and Cy5 median pixel intensity values obtained using Genepix 3.0 software (Axon, Foster City, CA) were background subtracted, and Cy3/Cy5 ratios were obtained. Cy3/Cy5 ratios were log-transformed (base 2) and hierarchically clustered (average linkage correlation metric) using the Cluster program from Stanford University [40]. Database calculations were performed and expression maps were generated with SAM (Significance Analysis of Microarrays) for Excel [41]. The Cy3/Cy5 ratios were compared between the PC3 and LNCaP cells in SAM using a one class analysis, with a two class analysis used for comparison of normal and malignant tissue samples. For the comparison of the RT-PCR data to the array results, the average fold differences for each gene were log-transformed (base 2) and compared to the similarly log-transformed (base 2) Cy3/Cy5 ratios obtained after Cluster and SAM analysis. Inter-sample comparison of the prostate and breast tumor miRNA profiles were visualized with Cluster and Java TreeView [42]. Genepix median of ratio values from experiments were log-transformed (base 2) and filtered for genes where data were present in 80% of experiments. Genes and arrays were hierarchically clustered, as described earlier, and miRNA expression heat maps were generated using SAM. The breast cancer miRNA heat map generated by unsupervised clustering analysis was subsequently labeled according to each cancer sample's clinical annotation for ErbB2 (+, -), ER (+, -), and PR (+, -) status. Fisher's Exact (2 × 2, 2 × 3) tests were performed to test the ability of the 2 and 3 most prominent dendrogram arms identified by unsupervised clustering to classify the set of breast cancer samples in accordance with their clinical annotation.

## Competing interests

The author(s) declare that they have no competing interests.

## Authors' contributions

MDM contributed to methods development, carried out the microarray studies, participated in design and coordination of the study, and drafted the manuscript. CMH participated in design and coordination of the study, assisted with analysis, and contributed to drafting the manuscript. VF provided the breast tumor samples and assisted with methods. CCB and GKS participated in study design and analysis, and contributed to drafting the manuscript. RCG contributed to methods development, participated in design and coordination of the study, and assisted in drafting the manuscript. JB and KS assisted with methods development, analysis, and microarray experiments. DGG and LW performed the qRT-PCR studies and performed data analysis. All authors read and approved the final manuscript.

## Supplementary Material

Additional File 1 Schematic of miRNA labeling procedure. Click here for file

Additional File 2 Fidelity of labeling and amplification procedures in microarray analysis. Click here for file

Additional File 3 Raw data of qRT-PCR and microarray data for PC3vs. LNCaP comparison. Click here for file

Additional File 4 miRNA expression in ErbB2+ tumors vs. SKBr3 cell line. Click here for file

Additional File 5 Clinical annotation for breast tumor specimens. Click here for file

Additional File 6 Oligonucleotide sequences printed on microarrays. Click here for file

Additional File 7 qRT-PCR data of let-7 and miR-16 in PC3 and LNCaP cells. Includes PCR efficiency data. Click here for file

Additional File 8 Raw data for microarrays of clinical prostate specimens Click here for file

Additional File 9 Raw data for microarrays of clinical breast tumor tissues. Click here for file
