# Supplementary figures and images for: Optimized high-throughput microRNA expression profiling provides novel biomarker assessment of clinical prostate and breast cancer biopsies
Source: Mol Cancer. 2006 Jun 19;5:24. doi: 10.1186/1476-4598-5-24 (PMC1563474; doi:10.1186/1476-4598-5-24)

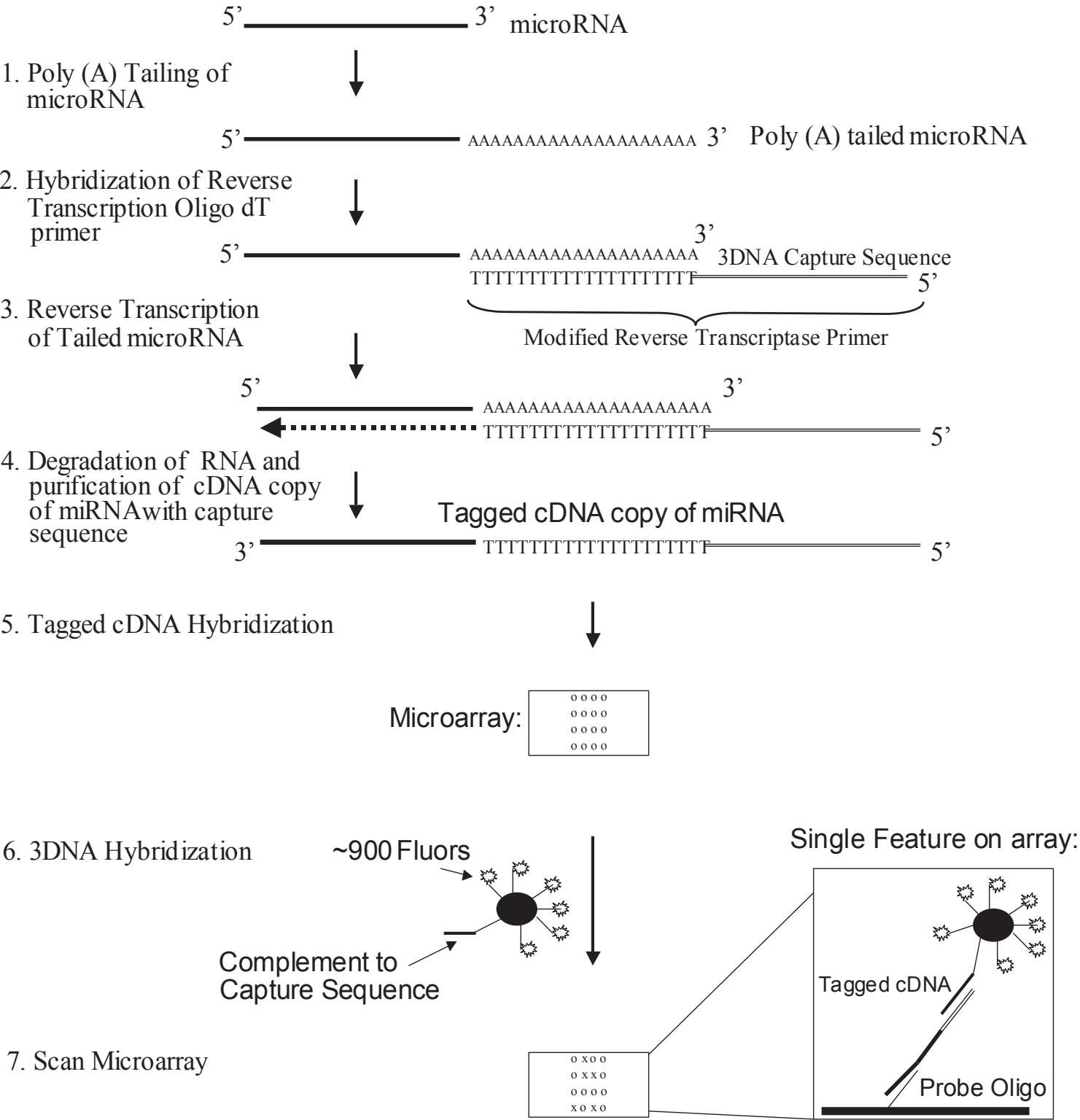

Supplement: Additional File 1 — Schematic of miRNA labeling procedure. [file 1476-4598-5-24-S1.pdf]
