# Supplementary material for: Optimized high-throughput microRNA expression profiling provides novel biomarker assessment of clinical prostate and breast cancer biopsies
Source: Mol Cancer. 2006 Jun 19;5:24. doi: 10.1186/1476-4598-5-24 (PMC1563474; doi:10.1186/1476-4598-5-24)
Supplement: Additional File 2 — Fidelity of labeling and amplification procedures in microarray analysis. [file 1476-4598-5-24-S2.doc]

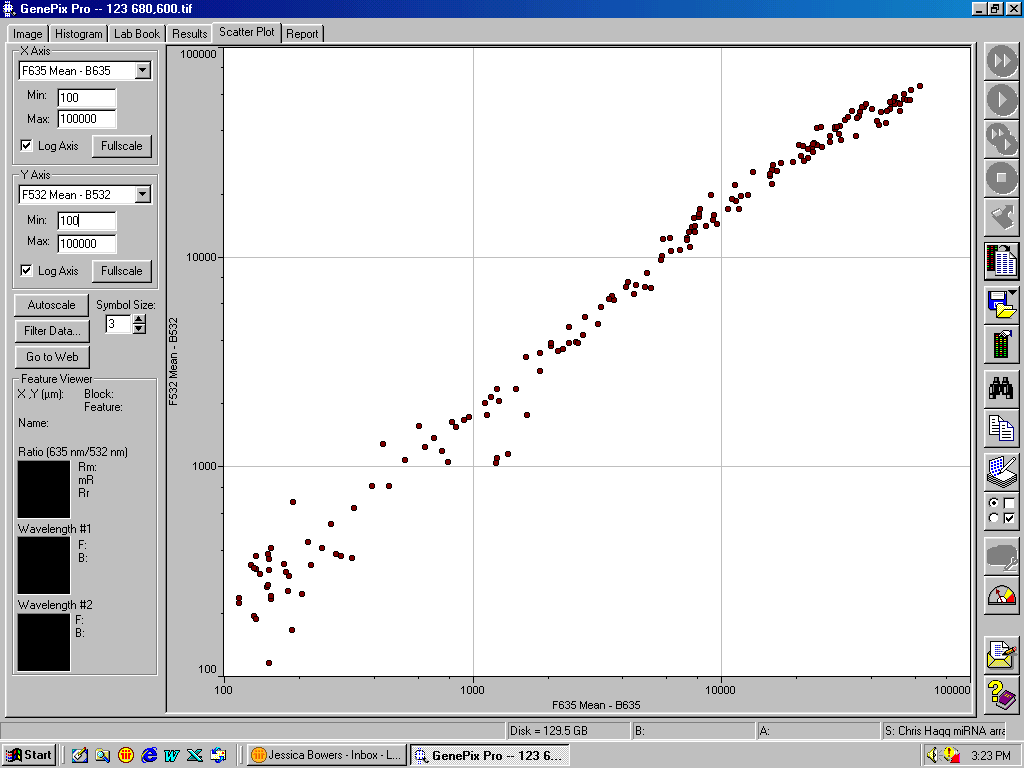
 R^2^ = 0.992

Equal amounts (150ng) of PC3 miRNA were labeled with Cy3 and Cy5 by the Array 900miRNA RT procedure. The Cy3 and Cy5 signal intensities were compared to determine the reproducibility of labeling the same sample in both channels.

Parallel labeling reactions were performed with 100ng of tumor (Cy5) and normal (Cy3) miRNA. The Cy3/Cy5 ratios were compared to determine the reproducibility of the labeling between the two arrays.

SenseAmp Plus miRNA Amplification

Amp. 1 vs. Amp. 2

y = 0.9612x - 40.195

R

2

= 0.9884

0

10000

20000

30000

40000

50000

60000

70000

0

10000

20000

30000

40000

50000

60000

70000

Amp 2 (s-b)

500 picograms of enriched miRNA was amplified in duplicate. Equal amounts (750ng) of both amplifications were labeled. The reproducibility of the amplification and labeling was determined by comparing the signal intensities or each of the features in both channels.

y = 1.1007x - 0.0447

R

2

2= 0.8751

-5

-4

-3

-2

-1

0

1

2

3

4

-4

-3

-2

-1

0

1

2

3

Amplified (Log_2_ Differential)

SenseAmp Plus miRNA Amplification

Unamplified vs Amplified Differential Analysis

Unamplified (Log_2_ Differential)

500 picograms of enriched Tumor and Normal miRNA was amplified. A fraction (1000ng) of each was labeled with either Cy3 (tumor) or Cy5 (normal). Parallel labeling reactions were performed using 150ng of unamplified miRNA from the same samples. The fidelity of the amplification and labeling was determined by comparing the differential expression of amplified to unamplified miRNAs.
