# Supplementary material for: Optimized high-throughput microRNA expression profiling provides novel biomarker assessment of clinical prostate and breast cancer biopsies
Source: Mol Cancer. 2006 Jun 19;5:24. doi: 10.1186/1476-4598-5-24 (PMC1563474; doi:10.1186/1476-4598-5-24)
Supplement: Additional File 4 — miRNA expression in ErbB2+ tumors vs. SKBr3 cell line. [file 1476-4598-5-24-S4.ppt]

## Slide 1
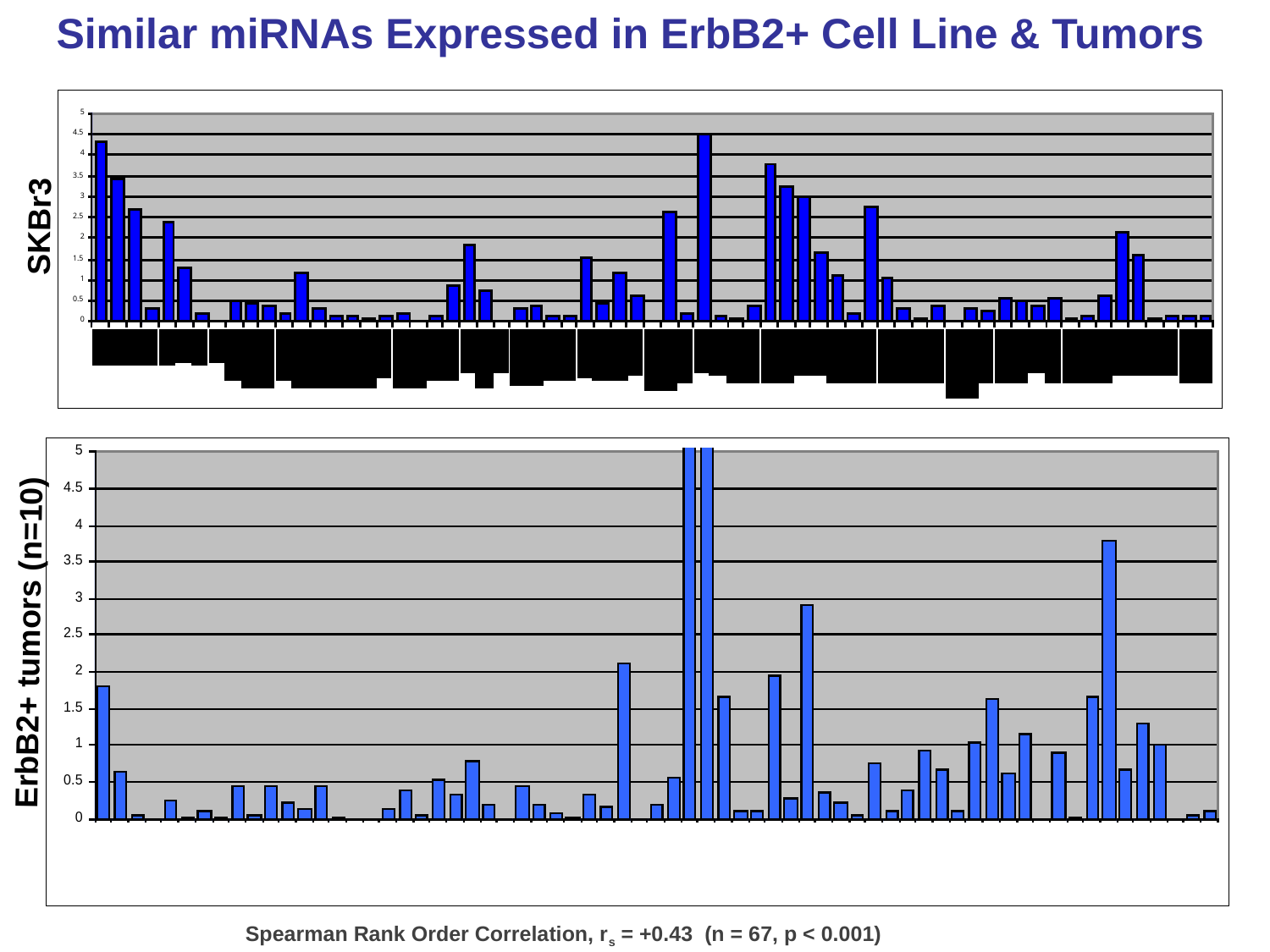

Similar miRNAs Expressed in ErbB2+ Cell Line & Tumors
SKBr3
ErbB2+ tumors (n=10)
Spearman Rank Order Correlation, rs = +0.43 (n = 67, p < 0.001)
